# Supplementary material for: Associations between handedness and brain functional connectivity patterns in children
Source: Nat Commun. 2024 Mar 15;15:2355. doi: 10.1038/s41467-024-46690-1 (PMC10943124; doi:10.1038/s41467-024-46690-1)
Supplement: Supplementary file 1 — Supplementary information [file 41467_2024_46690_MOESM1_ESM.docx]

**Associations between Handedness and Brain Functional Connectivity Patterns in Children**

Dardo Tomasi*^1^ and Nora D. Volkow^1^

^1^National Institute on Alcohol Abuse and Alcoholism, Bethesda, MD, 20892

**Supplementary Methods**

**Recruitment**

Details on recruitment strategies and inclusion and exclusion criteria for the ABCD study have been published and are available on the ABCD website (https://abcdstudy.org/scientists/protocols/). Briefly, the ABCD used probability sampling of U.S. schools within 21 catchment areas (geographical areas centered on schools within 50 miles of the research institution) as the primary method for contacting and recruiting eligible children and their parents^1^. Recruitment materials and electronic copies were provided to the families. Interested families completed a brief telephone screening and, if eligible, were enrolled and scheduled for the baseline assessment, which occurred at the research centers. Guardians and children were reimbursed for their participation. Recruitment closely represented demographic variables (sex, race, ethnicity, parental marital status and education, and income) of the general US population^2^.

**Inclusion and Exclusion criteria**

Children were not excluded unless they had severe psychiatric or neurological disorders or suffered from severe medical conditions. Specifically, exclusion criteria included common MRI contraindications, inability to understand or speak English fluently, uncorrected vision, hearing or sensorimotor impairments, a history of major neurological disorders, gestational age <28 weeks, birth weight <1,200 g, birth complications that resulted in hospitalization for more than 1 month, current diagnosis of schizophrenia, moderate or severe autism spectrum disorder, a history of traumatic brain injury or unwillingness to complete assessments^3,4^. ADHD children with or without medications were included.

**Sex, gender, and race**

Both girls (n=771) and boys (n=1029) participated in this study on handedness, ensuring representation from both sexes. Sex was defined at birth and was determined based on biological characteristics. Sex was considered as a covariate of no interest in the statistical analysis to account for any potential effects or differences related to sex. It is noted that the ABCD study, from which the data for this study were derived, collected sex and gender data, ensuring comprehensive data collection practices. Informed consent for sharing of individual-level data has been obtained by the ABCD study, adhering to ethical standards and privacy regulations. The lack of sex- and gender-based analysis in this study is due to the primary aim of the study, which was to investigate handedness without a specific focus on sex or gender differences. Sample size limitations may have precluded meaningful subgroup analyses.

Participants from diverse racial backgrounds, encompassing individuals from all races, were included in the ABCD study, ensuring representation across various racial groups. The classification of individuals into different race groups was based on self-reporting, allowing participants to identify their racial identity according to their own understanding and perception. Due to the limited sample size, we were unable to assess the specific effect of race on brain asymmetry comprehensively. However, race information was utilized as a covariate in statistical analyses to minimize potentially confounding effects of race on brain asymmetry.

**Supplementary Tables**

Supplementary Table S1: Locations of 10mm regions-of-interest (ROI) in the left (L) and right (R) brain hemispheres.

|  | ROI | Δ | |
| --- | --- | --- | --- |
| Label | **Side** | **Vertex #** |  |
| 6d | L | 5578 |  |
| FST | L | 23370 |  |
| BA1 | L | 7682 |  |
| 24dd | L | 4799 |  |
| POS2 | L | 12995 |  |
| BA4 | L | 4454 |  |
| BA40 | L | 17291 |  |
| IPS1 | L | 11977 |  |
| CER | L | 72407 |  |
| 6d | R | 5531 |  |
| FST | R | 23426 |  |
| BA1 | R | 7823 |  |
| 24dd | R | 4838 |  |
| POS2 | R | 13023 |  |
| BA4 | R | 4421 |  |
| BA40 | R | 17209 |  |
| IPS1 | R | 11871 |  |
| CER | R | 81504 |  |

6d: premotor area d; FST: fundus of the superior temporal visual area; CER: cerebellum lobe V; 24dd: mid cingulum; POS2: parieto-occipital sulcus, area 2; IPS1: intraparietal sulcus, area 1; BA1 somatosensory area; BA4: superior motor area, and BA40: inferior parietal area.

**Supplementary Figures**


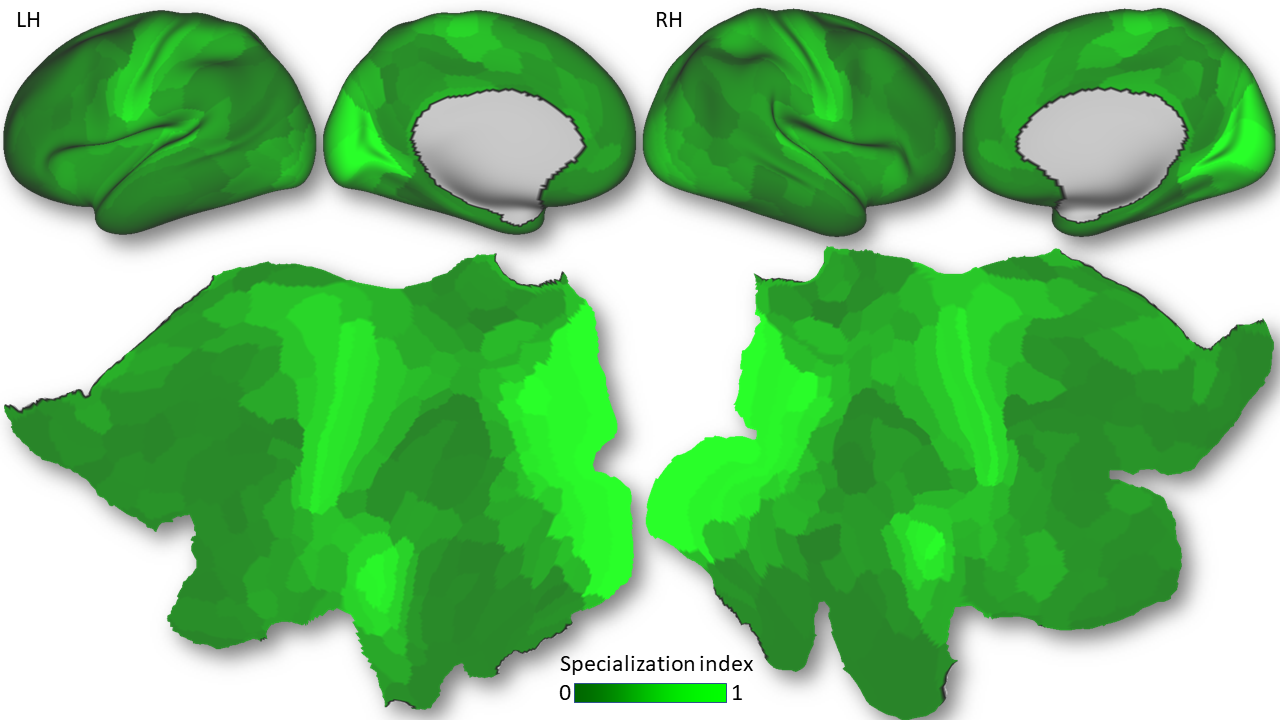


**Supplementary Fig. S1: Functional specialization index.** Overall functional specialization of the 360 ROIs in the multi-modal parcellation of human cerebral cortex^5^ overlaid on inflated lateral and medial surface (top) and flat views of the cerebral cortex.


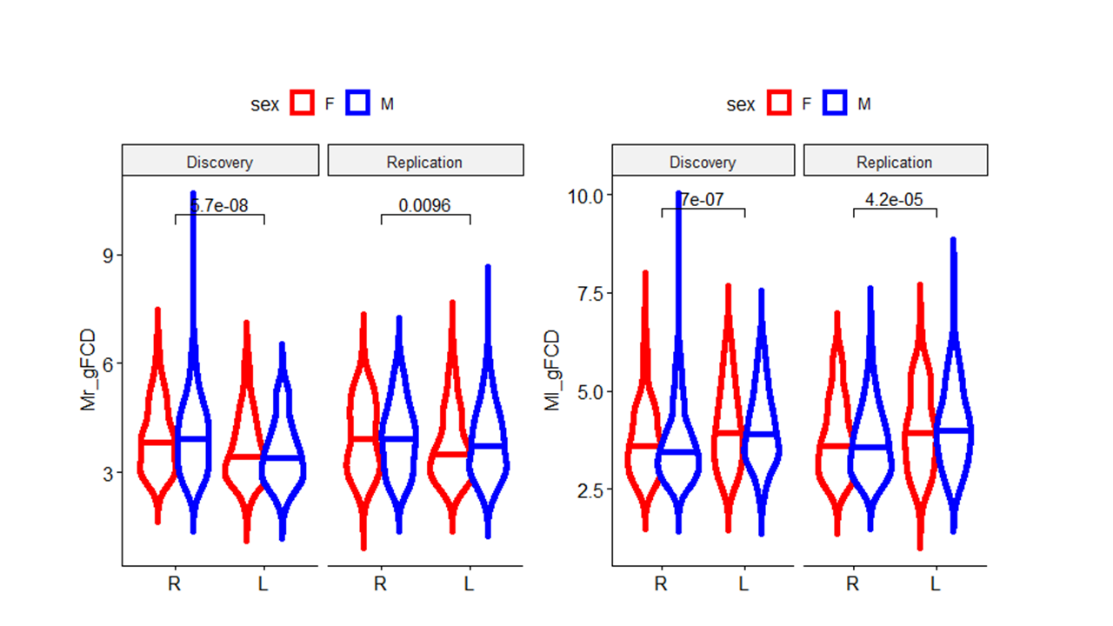


**Supplementary Fig. S2: Reproducibility of gFCD.** Global functional connectivity density (gFCD) in the right (Mr) ROI was lower and that in the left (Ml) ROI higher for 600 left-handers (L) than for 600 right-handers (R;), both in the Discovery (n=606) and Replication (n=594) subsamples but did not differ between 686 boys (M) and 514 girls (F).


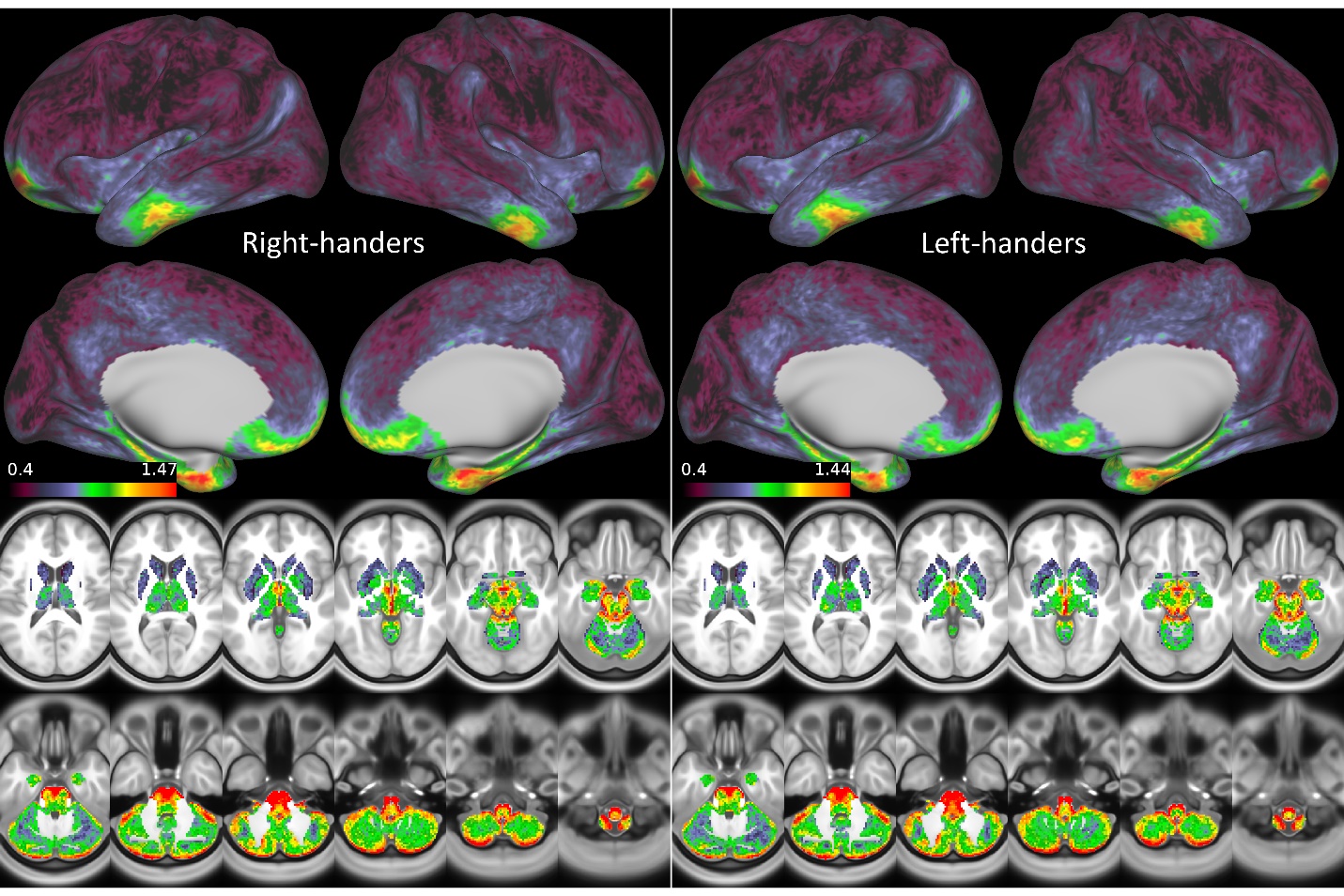


**Supplementary Fig. S3: Negative gFCD.** Average global functional connectivity density (gFCD) maps computed with a Pearson correlation threshold R < -0.6 across 600 right- and 600 left-handed children, overlaid on inflated lateral and medial surfaces of the left and right cerebral hemispheres and 12 axial views showing subcortical regions and cerebellum.


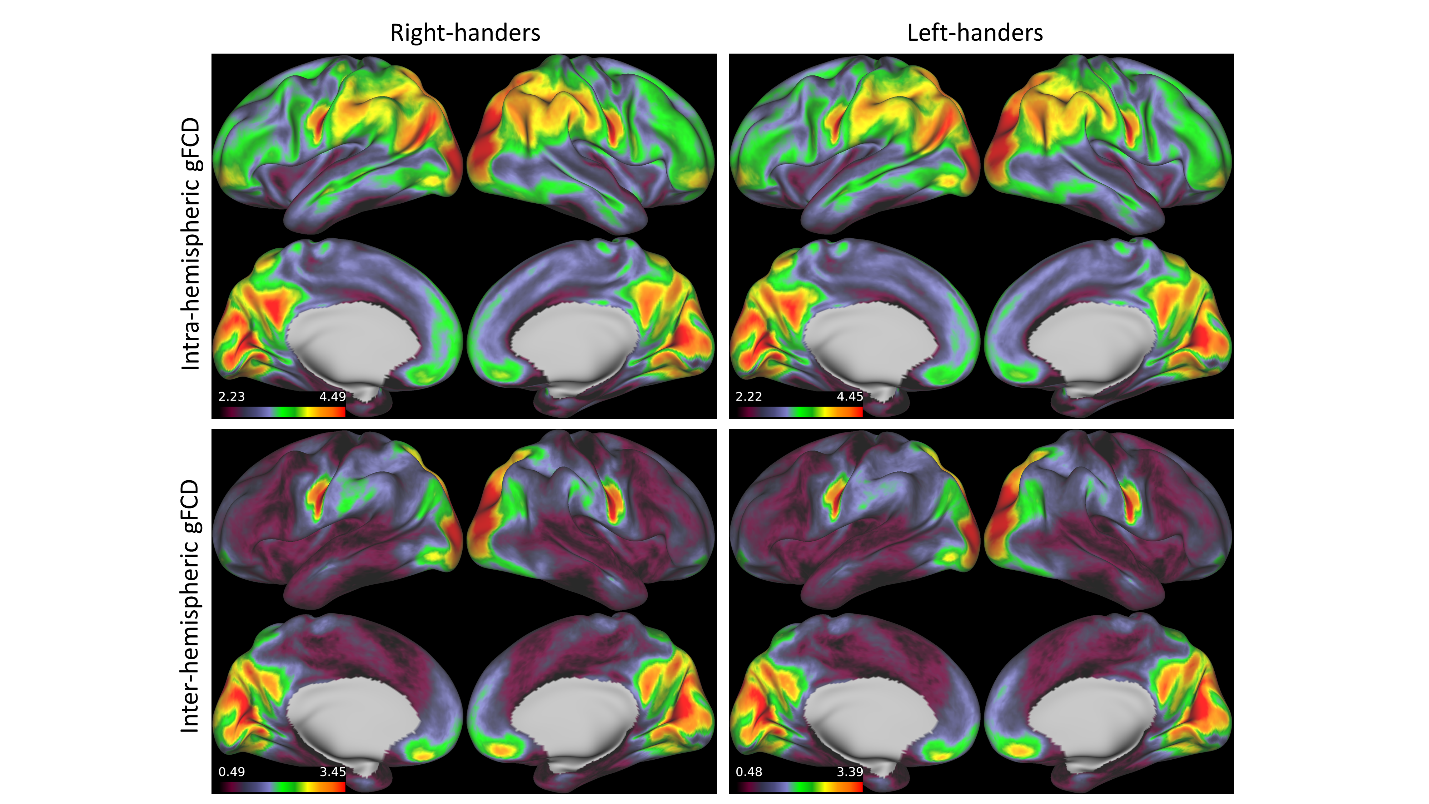


**Supplementary Fig. S4: Ipsilateral and contralateral gFCD patterns**. Average intra- and inter-hemispheric global functional connectivity density (gFCD) maps overlaid on inflated lateral and medial surfaces of the left and right cerebral hemispheres across 600 right- and 600 left-handed children.


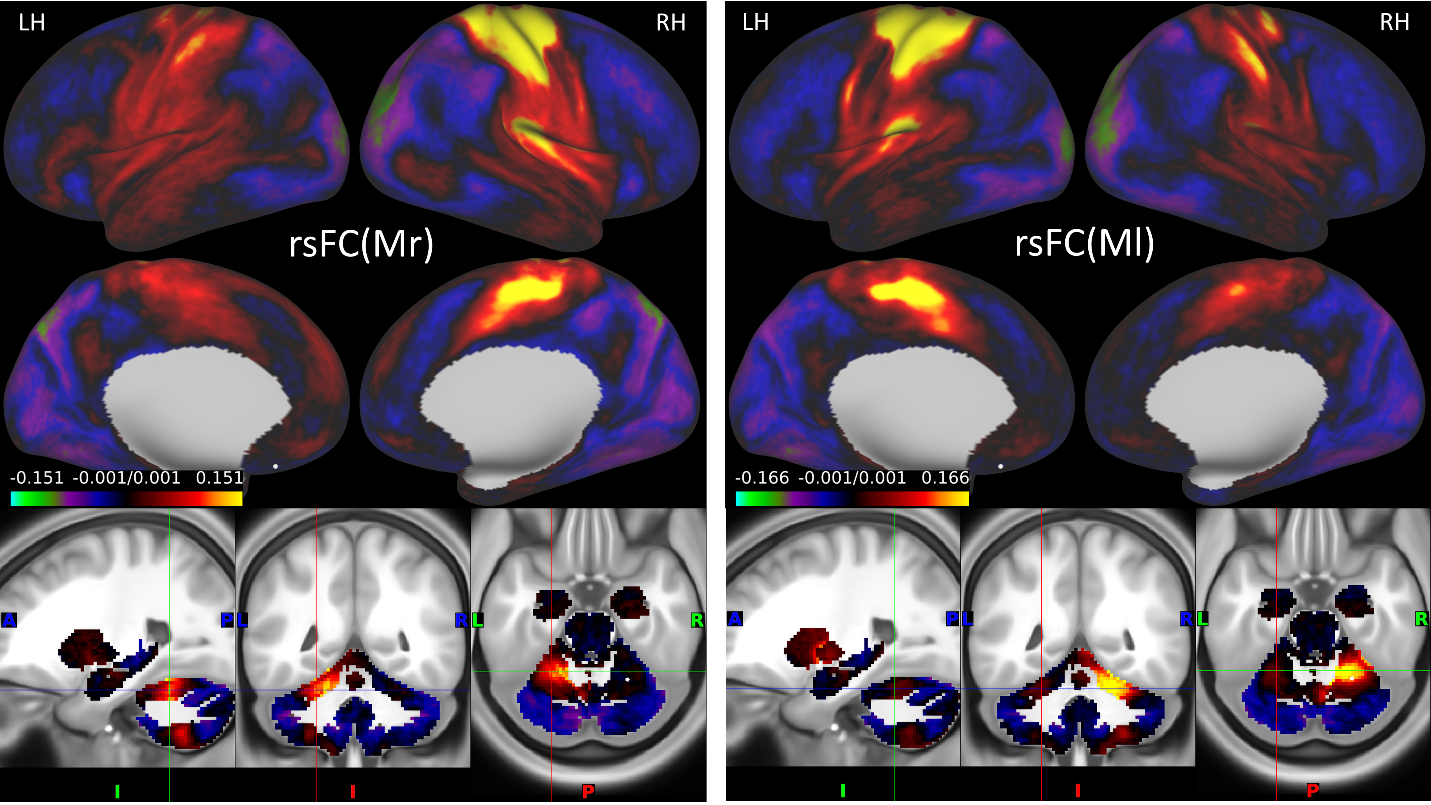


**Supplementary Fig. S5: Functional connectivity of the hand area.** Resting-state functional connectivity (rsFC; z-score maps) of the left (Ml) and right (Mr) seeds for 600 righthanders, superimposed on inflated lateral and ventral surfaces of the left (LH) and right (RH) hemispheres of the human cerebral cortex and 3 orthogonal brain views showing the connectivity patterns in subcortical regions and cerebellum.


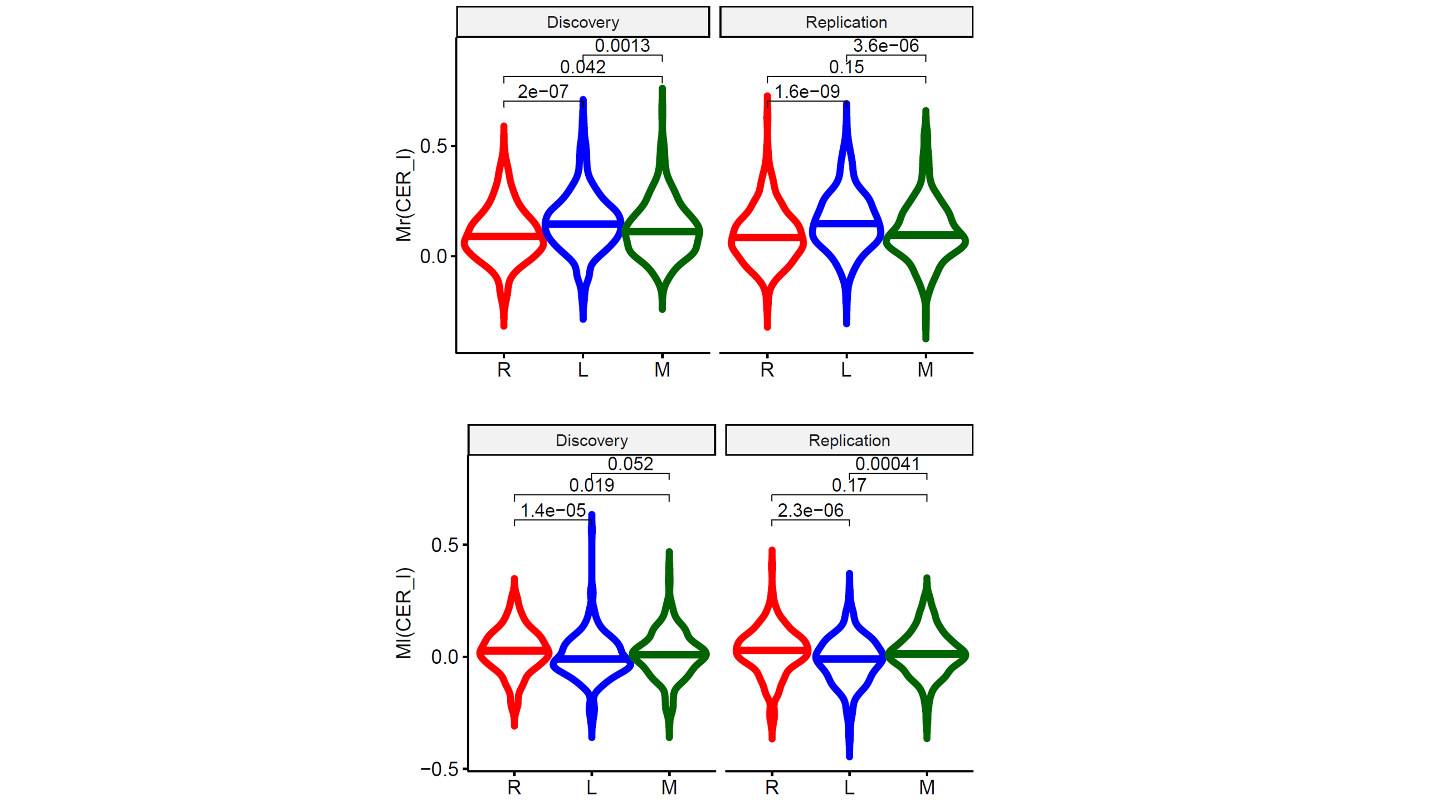
 **Supplementary Fig. S6: Connectivity of the hand motor area: Cerebellum.** Strength of the resting-state functional connectivity of left (Ml) and right (Mr) seeds in the left (CER_l) and right (CER_r) ROIs in cerebellum lobe V for 600 righthanders (R), 600 lefthanders (L), and 600 mixed handers.


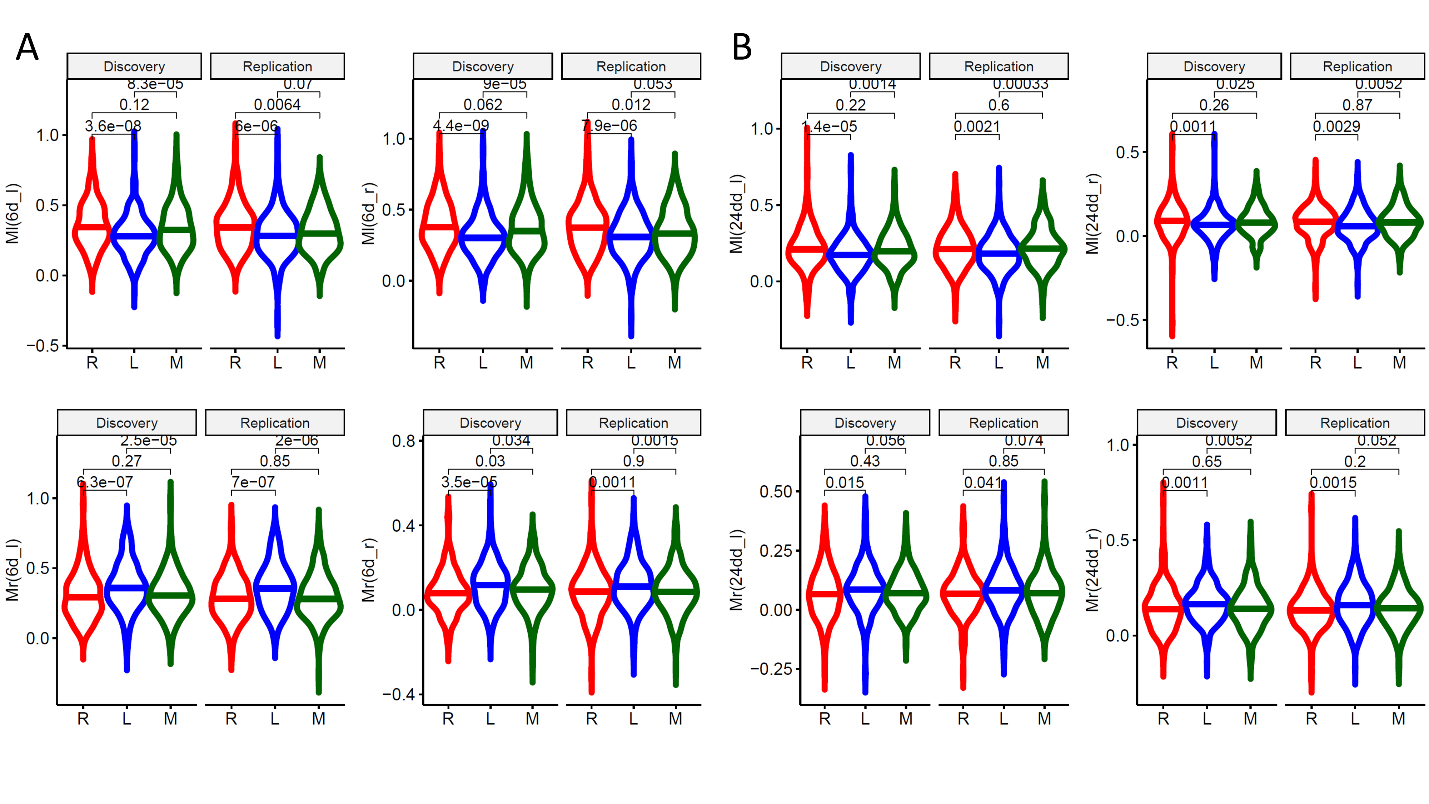
 **Supplementary Fig. S7: Connectivity of the hand motor area.** Strength of the resting-state functional connectivity of left (Ml) and right (Mr) seeds for left (_l) and right (_r) ROIs in premotor (6d; **A**) and mid cingulum (24dd; **B**) for 600 righthanders (R), 600 lefthanders (L), and 600 mixed handers.


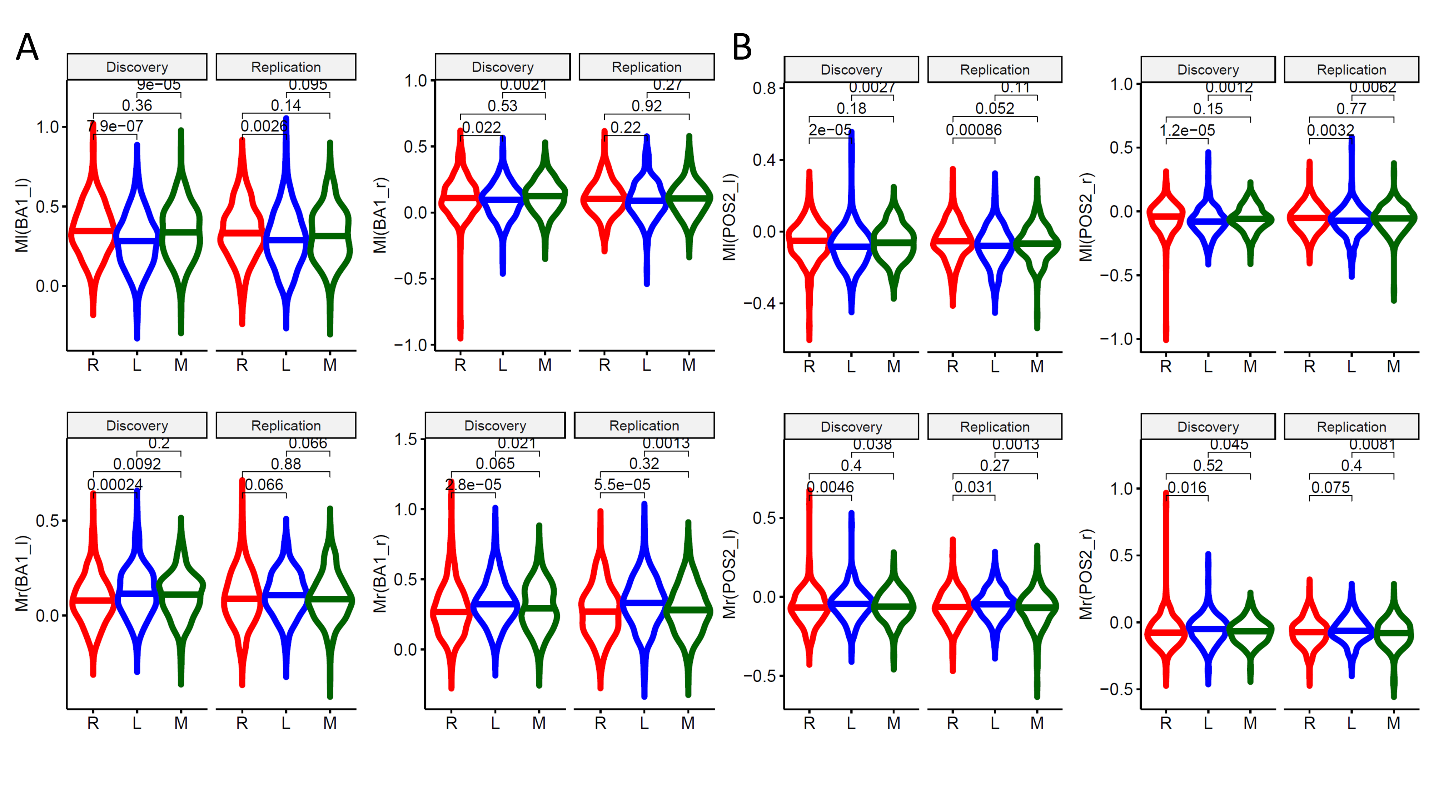
 **Supplementary Fig. S8: Connectivity of the hand motor area.** Strength of the resting-state functional connectivity of left (Ml) and right (Mr) seeds for left (_l) and right (_r) ROIs in somatosensory (BA1; **A**) and the parieto-occipital sulcus area 2 (POS2; **B**) for 600 righthanders (R), 600 lefthanders (L), and 600 mixed handers.


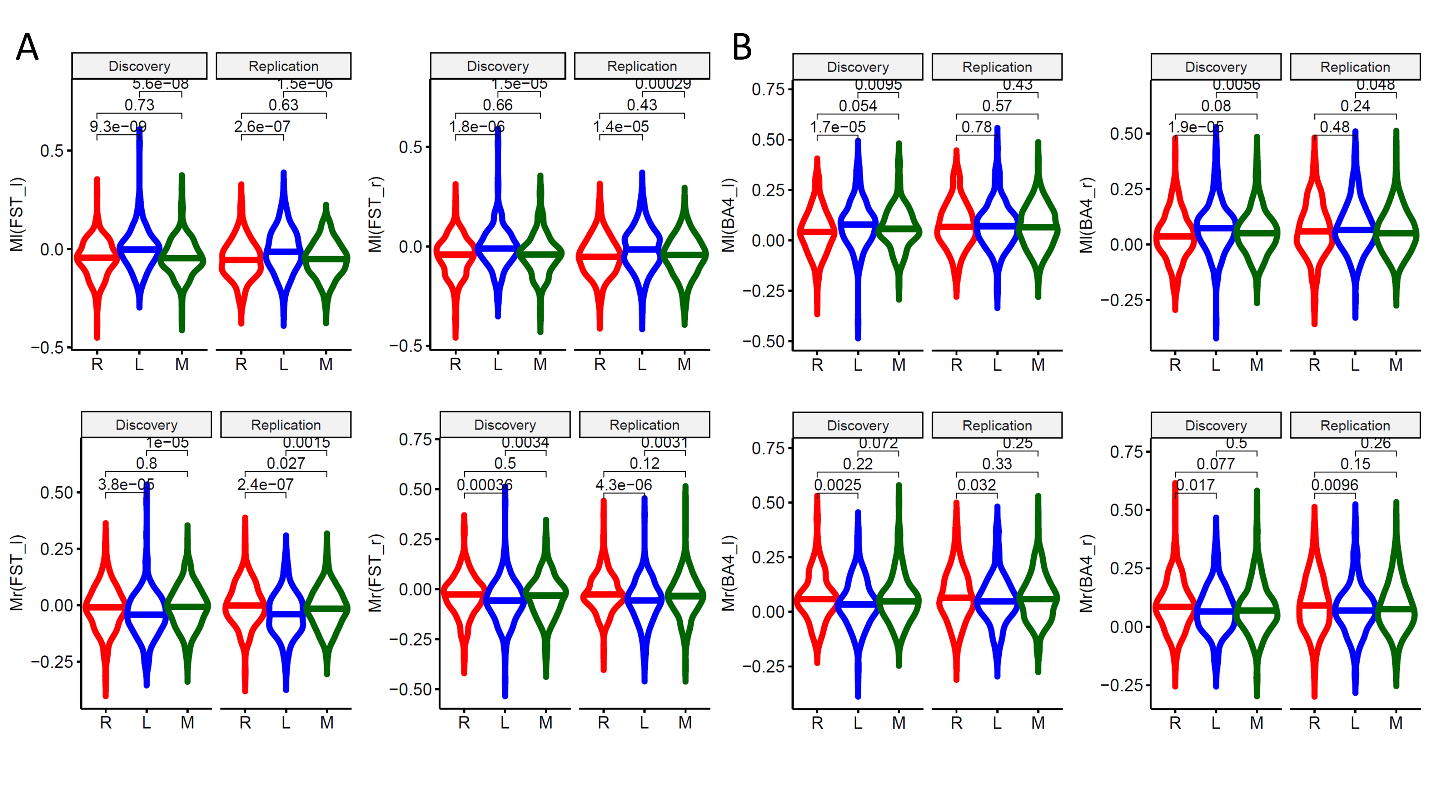


**Supplementary Fig. S9: Connectivity of the hand motor area.** Strength of the resting-state functional connectivity of left (Ml) and right (Mr) seeds for left (_l) and right (_r) ROIs in the fundus of the superior temporal visual area (FST; **A**) and the superior motor area (BA4; **B**) for 600 righthanders (R), 600 lefthanders (L), and 600 mixed handers.

**
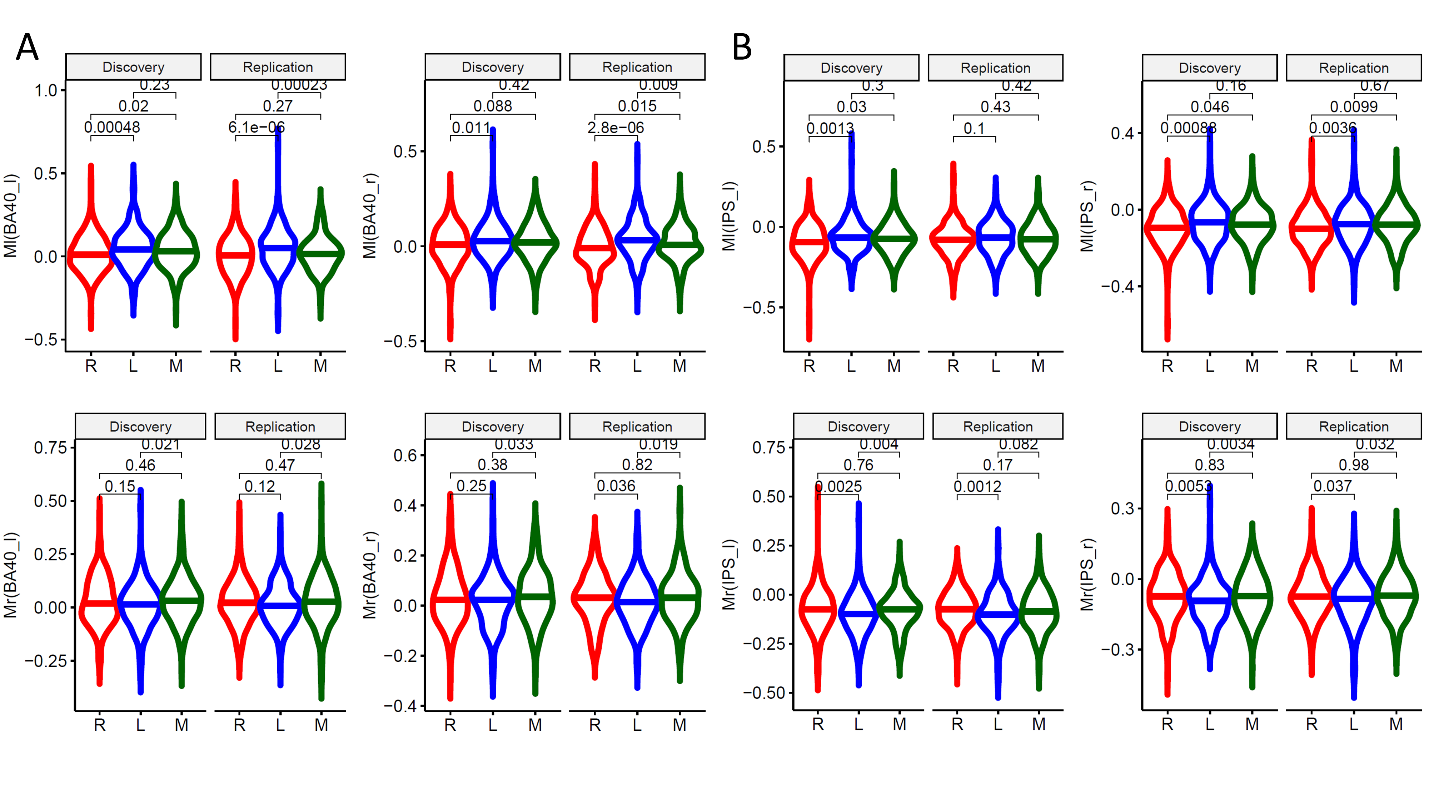
 Supplementary Fig. S10: Connectivity of the hand motor area.** Strength of the resting-state functional connectivity of left (Ml) and right (Mr) seeds for left (_l) and right (_r) ROIs in the inferior parietal cortex (BA40; **A**) and the intraparietal sulcus area 1 (IPS; **B**) for 600 righthanders (R), 600 lefthanders (L), and 600 mixed handers.

**`
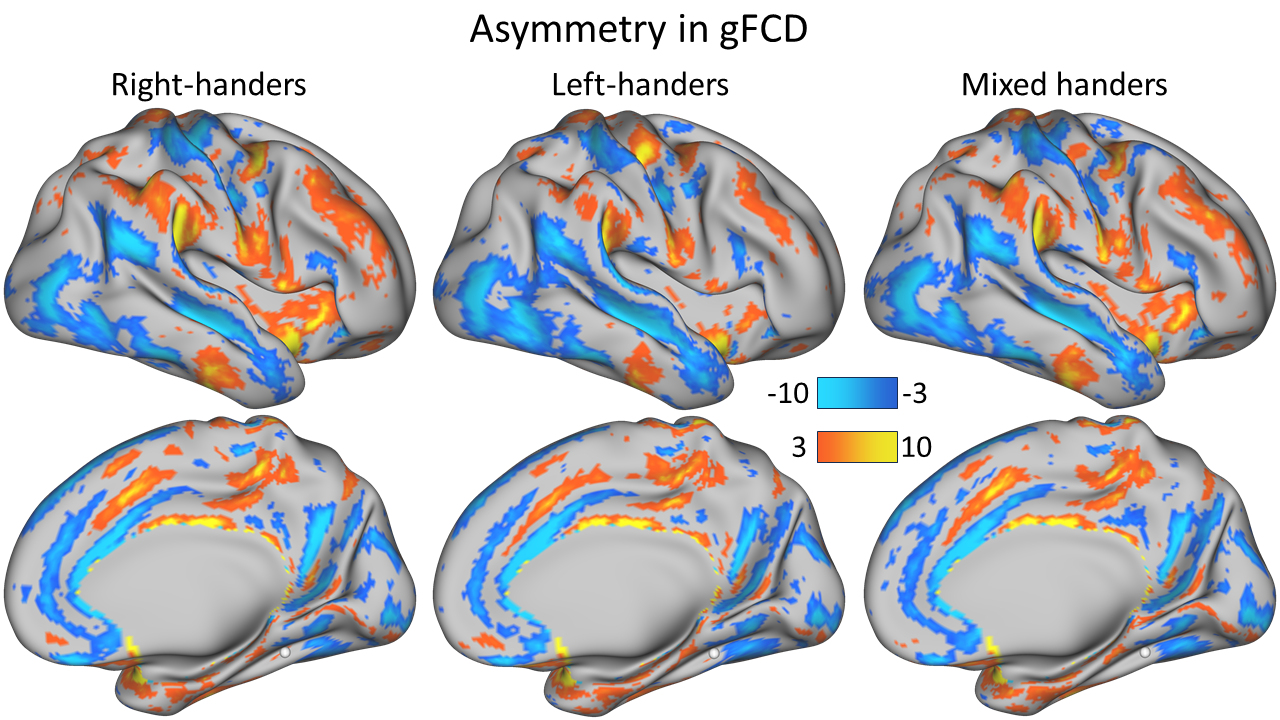
**

**Supplementary Fig. S11: Asymmetry in gFCD.** Statistical differences (t-scores) in global functional connectivity density (gFCD) between corresponding grayordinates in the right and left cortical hemispheres for 600 right-handers, 600 left-handers, and 600 mixed handers superimposed on lateral and medial views of the right cerebral hemisphere. Statistical model: One sample T-test. An FDR-corrected threshold P_FDR_<0.05 (2-sided) was used to display the statistical maps.

**
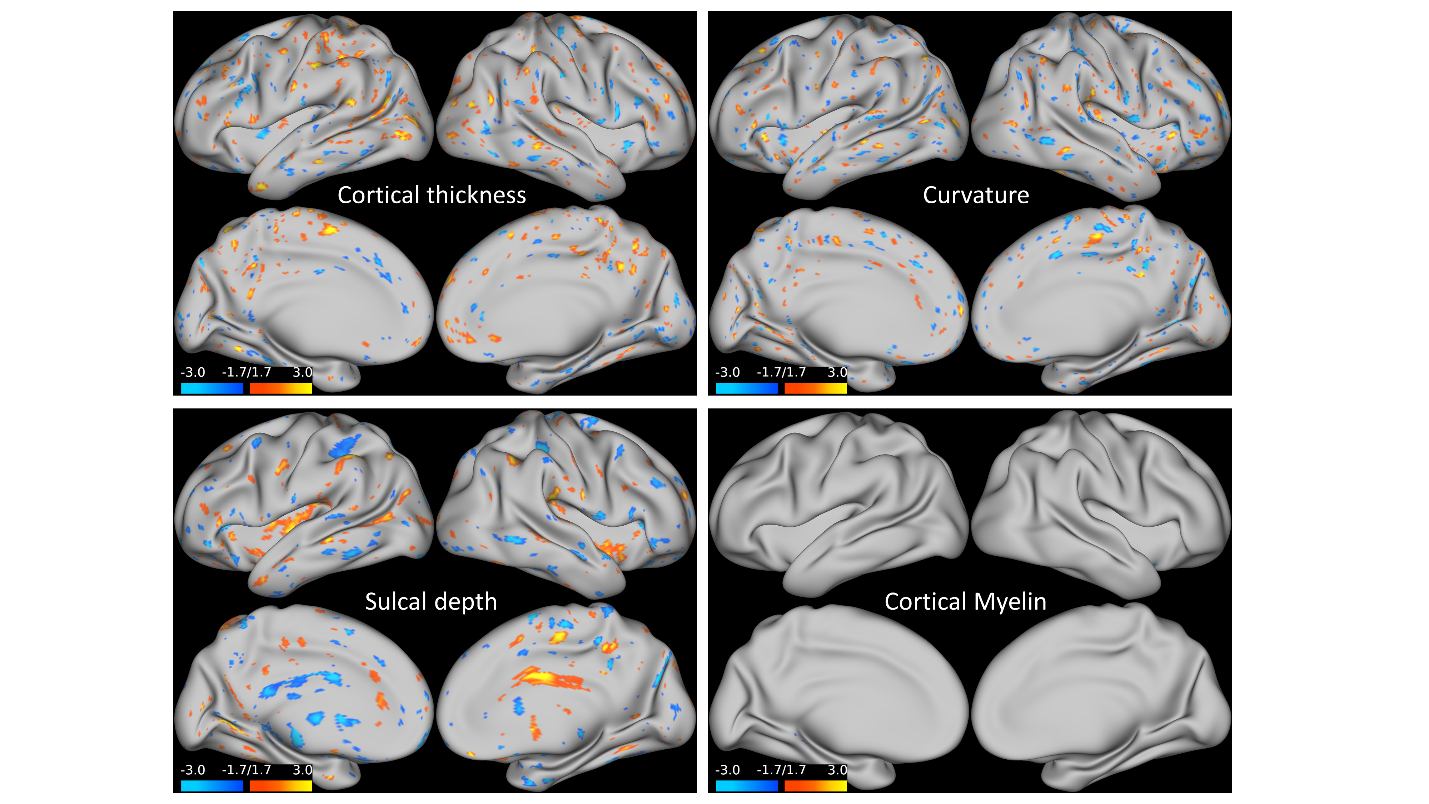
**

**Supplementary Fig. S12: Handedness and brain structure.** Statistical differences (t-scores) in cortical thickness, curvature, sulcal depth and cortical myelin between 600 right-handers, 600 left-handers, superimposed on lateral and medial views of the cerebral hemispheres. Statistical model: two sample T-test. An uncorrected threshold P<0.05 (2-sided) was used to display the statistical maps.

**
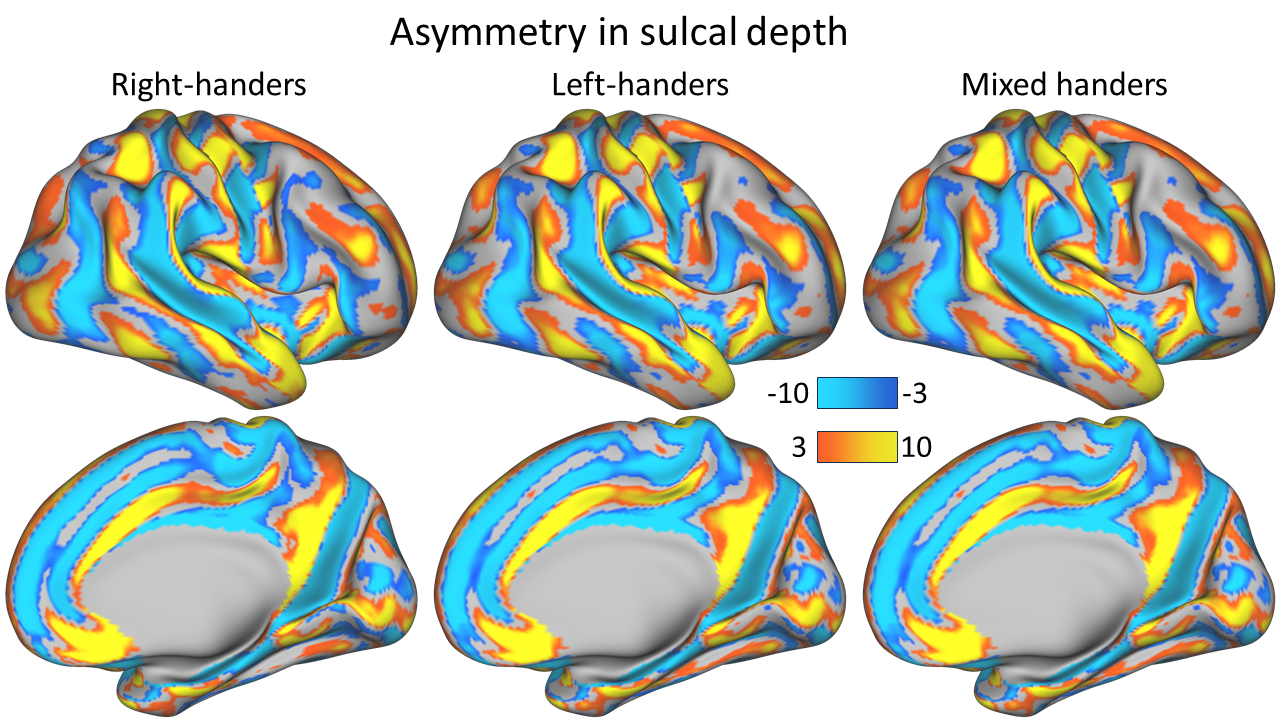
**

**Supplementary Fig. S13: Asymmetry in sulcal depth.** Statistical differences (t-scores) in sulcal depth between corresponding grayordinates in the right and left cortical hemispheres for 600 right-handers, 600 left-handers, and 600 mixed handers superimposed on lateral and medial views of the right cerebral hemisphere. Statistical model: One sample T-test. An FDR-corrected threshold P_FDR_<0.05 (2-sided) was used to display the statistical maps.

**
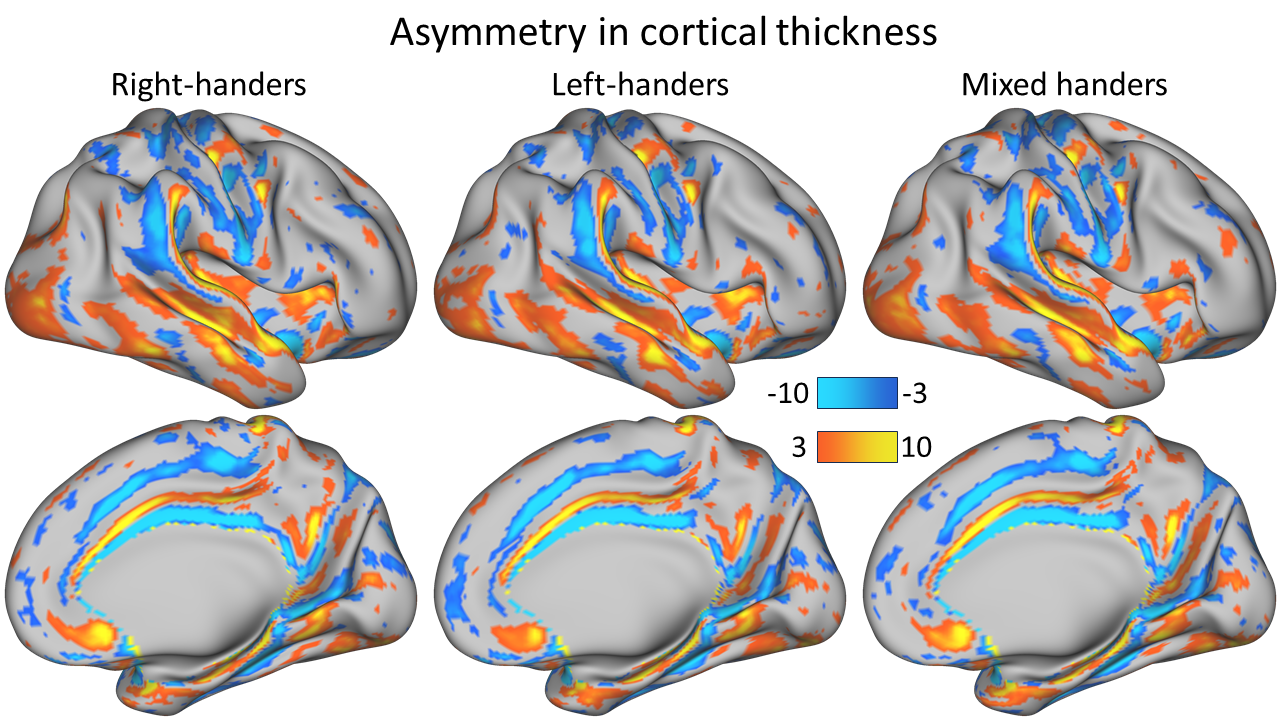
**

**Supplementary Fig. S14: Asymmetry in cortical thickness.** Statistical differences (t-scores) in cortical thickness between corresponding grayordinates in the right and left cortical hemispheres for 600 right-handers, 600 left-handers, and 600 mixed handers superimposed on lateral and medial views of the right cerebral hemisphere. Statistical model: One sample T-test. An FDR-corrected threshold P_FDR_<0.05 (2-sided) was used to display the statistical maps.

**
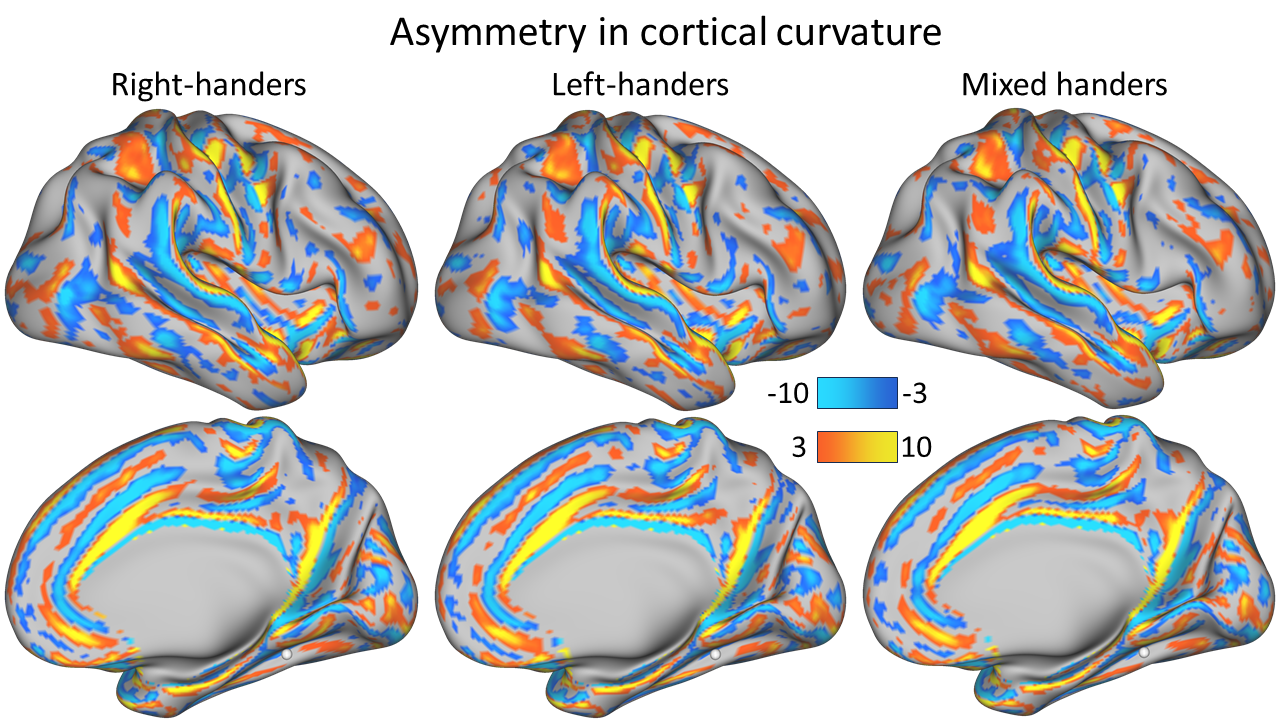
**

**Supplementary Fig. S15: Asymmetry in cortical curvature.** Statistical differences (t-scores) in cortical curvature between corresponding grayordinates in the right and left cortical hemispheres for 600 right-handers, 600 left-handers, and 600 mixed handers superimposed on lateral and medial views of the right cerebral hemisphere. Statistical model: One sample T-test. An FDR-corrected threshold P_FDR_<0.05 (2-sided) was used to display the statistical maps.

**
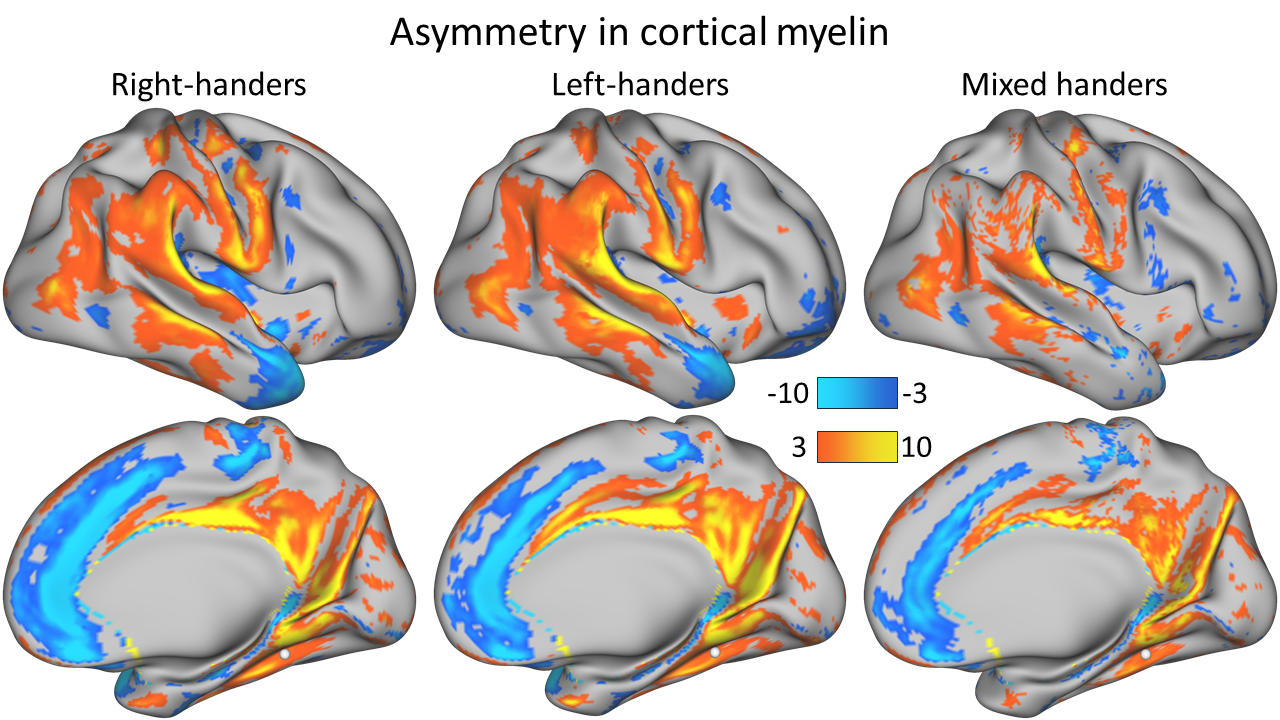
 Supplementary Fig. S16: Asymmetry in cortical myelin.** Statistical differences (t-scores) in cortical myelin between corresponding grayordinates in the right and left cortical hemispheres for 600 right-handers, 600 left-handers, and 600 mixed handers superimposed on lateral and medial views of the right cerebral hemisphere. Statistical model: One sample T-test. An FDR-corrected threshold P_FDR_<0.05 (2-sided) was used to display the statistical maps.

**
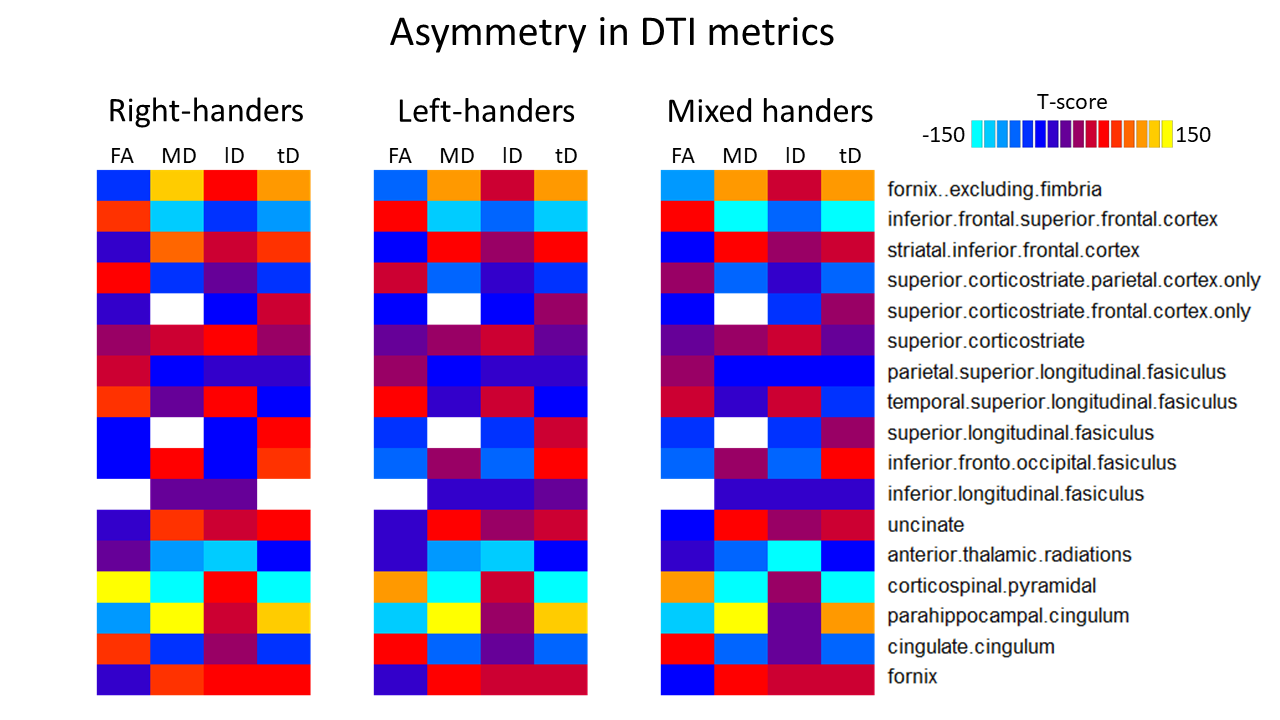
**

**Supplementary Fig. S17: Asymmetry in white matter diffusion metrics.** Statistical differences (t-scores) in fractional anisotropy (FA) and mean (MD), longitudinal (lD), and transverse (tD) diffusivities between the right and left cerebral hemispheres for 17 white matter fiber bundles across 392 right-handers, 392 left-handers, and 393 mixed handers. Statistical model: One sample T-test. An FDR-corrected threshold P_FDR_<0.05 (2-sided) was used to display the statistical maps.

**
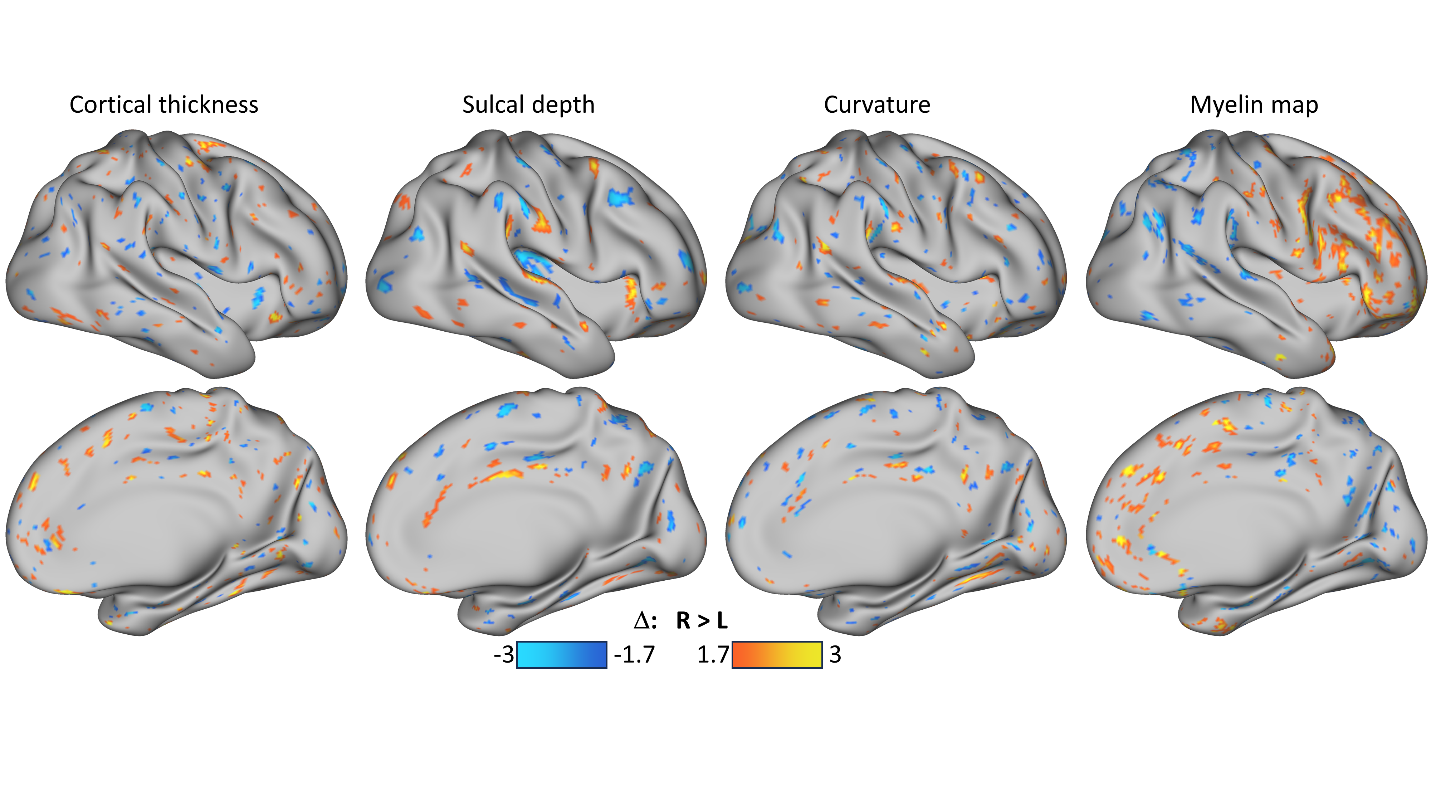
**

**Supplementary Fig. S18: Handedness and structural asymmetry.** Statistical differences (t-scores) in asymmetry for cortical thickness, sulcal depth, curvature, and cortical myelin between 600 right-handers, 600 left-handers, superimposed on lateral and medial views of the right cerebral surface. Statistical model: two sample T-test. An uncorrected threshold P<0.05 (2-sided) was used to display the statistical maps.


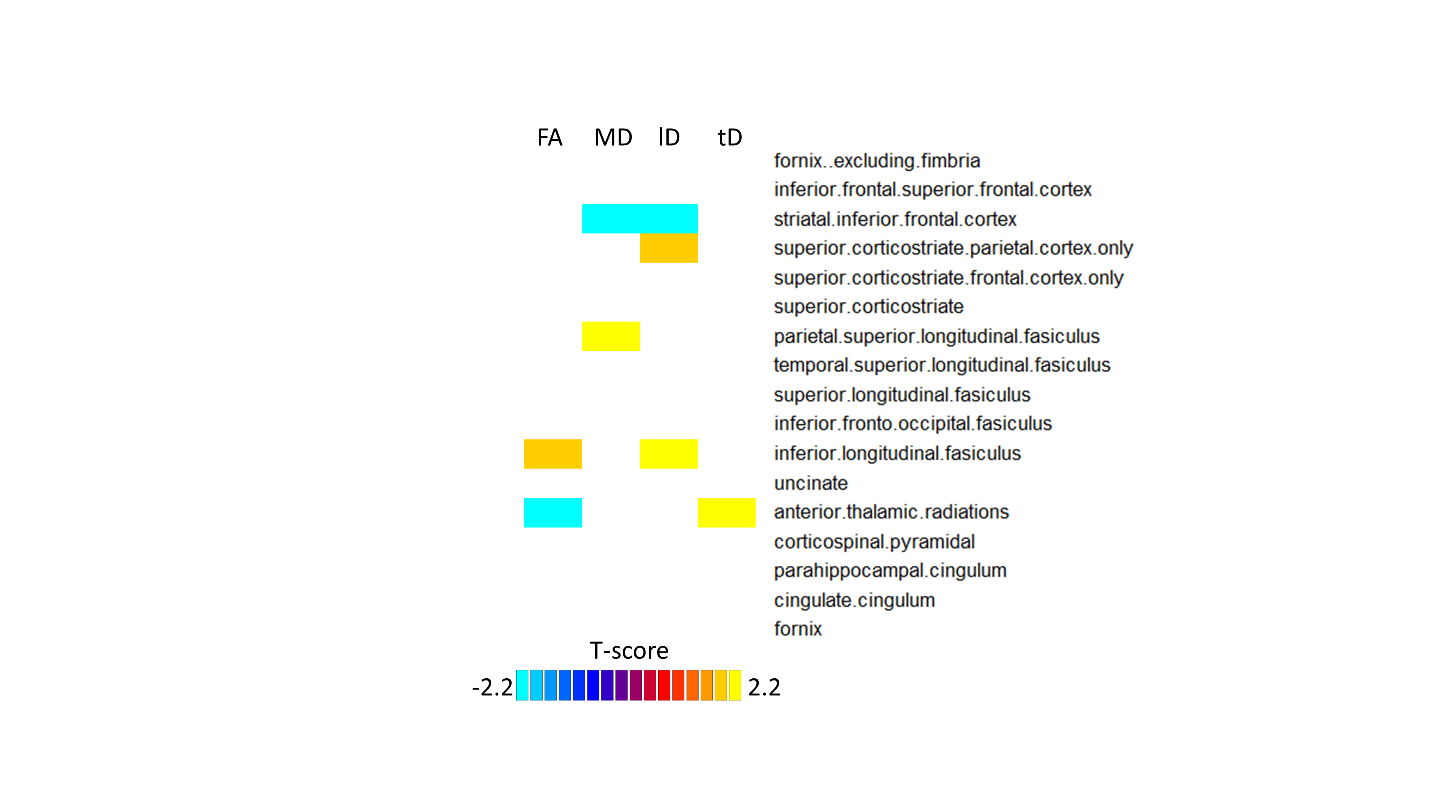


**Supplementary Fig. S19: Handedness and white matter diffusion asymmetry.** Statistical differences (t-scores) in interhemispheric asymmetry for fractional anisotropy (FA) as well as mean (MD), longitudinal (lD), and transverse (tD) diffusivities for 17 white matter fiber bundles across 392 right-handers, 392 left-handers. Statistical model: One sample T-test. An uncorrected threshold P<0.05 (2-sided) was used for display.

**
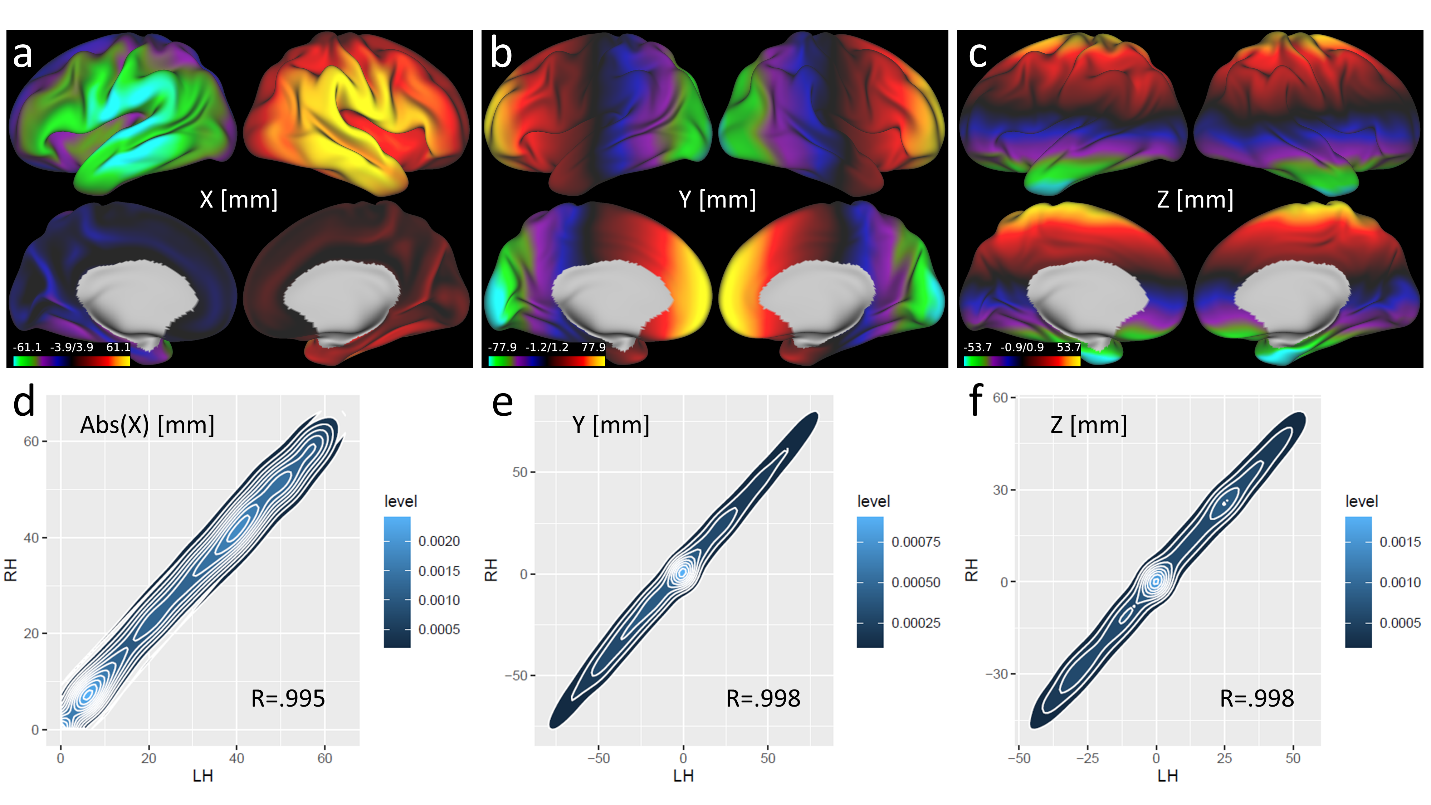
**

**Supplementary Fig. S20: Interhemispheric vertex correspondence.** Cartesian coordinates of 64,894 vertices on the cerebral surface (**a**-**c**). 2d-density plots showing the correspondence of these coordinates in the left (LH) and right (RH) hemispheres (d-f).

**Supplementary References**

1. Garavan, H.*, et al.* Recruiting the ABCD sample: Design considerations and procedures. *Dev Cogn Neurosci* **32**, 16-22 (2018).

2. Thompson, W.*, et al.* The structure of cognition in 9 and 10 year-old children and associations with problem behaviors: Findings from the ABCD study’s baseline neurocognitive battery. *Dev Cogn Neurosci* **36**, 100606 (2019).

3. Jernigan, T. & Brown, S. Introduction. *Dev Cogn Neurosci* **32**, 1-3 (2018).

4. Karcher, N.*, et al.* Assessment of the Prodromal Questionnaire-Brief Child Version for Measurement of Self-reported Psychoticlike Experiences in Childhood. *JAMA Psychiatry* **75**, 853-861 (2018).

5. Glasser, M.*, et al.* A multi-modal parcellation of human cerebral cortex. *Nature* **536**, 171-178 (2016).
