# Supplementary material for: Associations between handedness and brain functional connectivity patterns in children
Source: Nat Commun. 2024 Mar 15;15:2355. doi: 10.1038/s41467-024-46690-1 (PMC10943124; doi:10.1038/s41467-024-46690-1)
Supplement: Supplementary file 3 — Reporting Summary [file 41467_2024_46690_MOESM3_ESM.pdf]

nature portfolio

Corresponding author(s): Dardo Tomasi

Last updated by author(s): Feb 27, 2024

Reporting Summary

Nature Portfolio wishes to improve the reproducibility of the work that we publish. This form provides structure for consistency and transparency in reporting. For further information on Nature Portfolio policies, see our Editorial Policies and the Editorial Policy Checklist.

Statistics

For all statistical analyses, confirm that the following items are present in the figure legend, table legend, main text, or Methods section.

n/a	Confirmed	
☐	☒	The exact sample size (n) for each experimental group/condition, given as a discrete number and unit of measurement
☐	☒	A statement on whether measurements were taken from distinct samples or whether the same sample was measured repeatedly
☐	☒	The statistical test(s) used AND whether they are one- or two-sided<br>*Only common tests should be described solely by name; describe more complex techniques in the Methods section.*
☐	☒	A description of all covariates tested
☐	☒	A description of any assumptions or corrections, such as tests of normality and adjustment for multiple comparisons
☐	☒	A full description of the statistical parameters including central tendency (e.g. means) or other basic estimates (e.g. regression coefficient) AND variation (e.g. standard deviation) or associated estimates of uncertainty (e.g. confidence intervals)
☐	☒	For null hypothesis testing, the test statistic (e.g. F, t, r) with confidence intervals, effect sizes, degrees of freedom and P value noted<br>*Give P values as exact values whenever suitable.*
☒	☐	For Bayesian analysis, information on the choice of priors and Markov chain Monte Carlo settings
☒	☐	For hierarchical and complex designs, identification of the appropriate level for tests and full reporting of outcomes
☐	☒	Estimates of effect sizes (e.g. Cohen's d, Pearson's r), indicating how they were calculated

Our web collection on statistics for biologists contains articles on many of the points above.

Software and code

Policy information about availability of computer code

Data collection	The data used for this study was collected by the ABCD consortium. As part of the consortium's data collection procedures, the specific software tools and code implementations utilized in data acquisition and processing are not disclosed to external parties. Therefore, detailed information regarding the software used by the ABCD study is not available to us.
Data analysis	MATLAB R2023a; R4.0; RStudio 2023.03.0

For manuscripts utilizing custom algorithms or software that are central to the research but not yet described in published literature, software must be made available to editors and reviewers. We strongly encourage code deposition in a community repository (e.g. GitHub). See the Nature Portfolio guidelines for submitting code & software for further information.

Data

Policy information about availability of data

All manuscripts must include a data availability statement. This statement should provide the following information, where applicable:

- Accession codes, unique identifiers, or web links for publicly available datasets
- A description of any restrictions on data availability
- For clinical datasets or third party data, please ensure that the statement adheres to our policy

ABCD data are publicly available through the National Institute of Mental Health Data Archive (https://nda.nih.gov). The HCP S1200 average task-fMRI Cohen's d effect-size maps across 997 subjects in CIFTI format were downloaded from the Brain Analysis Library of Spatial Maps and Atlases (https://balsa.wustl.edu/).

Research involving human participants, their data, or biological material

Policy information about studies with human participants or human data. See also policy information about sex, gender (identity/presentation), and sexual orientation and race, ethnicity and racism.

Reporting on sex and gender	Findings from this study do not apply exclusively to one sex, as both girls and boys were included in the analysis. Specifically, both girls (n=771) and boys (n=1029) participated in this study on handedness, ensuring representation from both sexes. Sex was defined at birth and was determined based on biological characteristics. Sex was considered as a covariate of no interest in the statistical analysis to account for any potential effects or differences related to sex. It is noted that the ABCD study, from which the data for this study were derived, collected sex and gender data, ensuring comprehensive data collection practices. Informed consent for sharing of individual-level data has been obtained by the ABCD study, adhering to ethical standards and privacy regulations. The lack of sex- and gender-based analysis in this study is due to the primary aim of the study, which was to investigate handedness without a specific focus on sex or gender differences. Sample size limitations may have precluded meaningful subgroup analyses based on sex or gender.
Reporting on race, ethnicity, or other socially relevant groupings	Participants from diverse racial backgrounds, encompassing individuals from all races, were included in the ABCD study, ensuring representation across various racial groups. The classification of individuals into different race groups was based on self-reporting, allowing participants to identify their racial identity according to their own understanding and perception. Due to the limited sample size, we were unable to assess the specific effect of race on brain asymmetry comprehensively. However, race information was utilized as a covariate in statistical analyses to minimize potentially confounding effects of race on brain asymmetry.
Population characteristics	We studied 9-10 years old children with demographic characteristics of the general US population. Children were fluent in English and excluded if they had medical, neurological, or cognitive problems, poor English-language proficiency, or contraindications for MRI.
Recruitment	Details on recruitment strategies and inclusion and exclusion criteria for the ABCD study have been published and are available on the ABCD website (https://abcdstudy.org/scientists/protocols/). Briefly, the ABCD used probability sampling of U.S. schools within 21 catchment areas (geographical areas centered on schools within 50 miles of the research institution) as the primary method for contacting and recruiting eligible children and their parents. Recruitment materials and electronic copies were provided to the families. Interested families completed a brief telephone screening and, if eligible, were enrolled and scheduled for the baseline assessment, which occurred at the research centers. Guardians and children were reimbursed for their participation. Recruitment closely represented demographic variables (sex, race, ethnicity, parental marital status and education, and income) of the general US population.
Ethics oversight	The ABCD Study was approved by the institutional review board (IRB) at the University of California in San Diego and obtained local IRB approval at 21 data collection sites across the United States

Note that full information on the approval of the study protocol must also be provided in the manuscript.

Field-specific reporting

Please select the one below that is the best fit for your research. If you are not sure, read the appropriate sections before making your selection.

☒ Life sciences ☐ Behavioural & social sciences ☐ Ecological, evolutionary & environmental sciences

For a reference copy of the document with all sections, see nature.com/documents/nr-reporting-summary-flat.pdf

Life sciences study design

All studies must disclose on these points even when the disclosure is negative.

Sample size	600 right-handed, 600 left-handed, and 600 mided handed children matched by by age, sex, race, scanner, and total brain volume.We aimed to detect small effects in brain connectivity between left-handed and right-handed individuals. We had access to imaging data from 600 left-handers from the ABCD study. We employed a rigorous matching procedure to select 600 right-handers and 600 mixed-handers from the larger ABCD sample. Matching by age, sex, race, scanner, and total brain volume helped control for potential confounding variables and ensured comparability between the groups. Given these considerations, our sample size of 600 left-handers and 600 right-handers was deemed sufficient to detect differences in brain connectivity of small effect size. Specifically, with a Cohen's d of 0.21, we anticipated that our study would have adequate power to detect these small effects. This sample size provides confidence in the robustness and generalizability of our findings regarding the neural correlates of handedness.
Data exclusions	Participants with excessive levels of head motion during resting-state fMRI (>50% of time points with framewise displacement (FD)<0.Smm) were not included.
Replication	Participants were split into 2 independent demographically matched subsamples: Discovery (N=909, 303 left-, 303 right-, and 303 mixed-handed) and Replication (N=891, 297 left-, 297 right-, and 297 mixed-handed) . There were no significant differences in brain volume, age, and FD, and in the proportions of MRI manufacturers and race/ethnicity groups between the Discovery and Replication subsamples. All

attempts to replication were successful.

Randomization

The sample randomization method was used. Specifically, participants were randomly assigned to Discovery and Replication subsamples using ABCC's "matched group" status, which is based on sociodemographic factors that can impact brain development, such as age, sex, ethnicity, grade, the highest level of parental education, and handedness.

Blinding

Blinding of participants or researchers was not feasible because handedness was self-reported by participants based on their hand preference. Participants are typically aware of their own handedness and blinding them to this information was impractical. Attempting to blind participants in a handedness study may introduce unnecessary complexity and could potentially compromise the validity of the study. The assessment of handedness is generally straightforward and unlikely to be influenced by bias or subjective interpretation. Therefore, the lack of blinding is unlikely to introduce significant bias into the study findings. Furthermore, handedness is a well-established and widely studied trait, and previous research has often been conducted without blinding.

Reporting for specific materials, systems and methods

We require information from authors about some types of materials, experimental systems and methods used in many studies. Here, indicate whether each material, system or method listed is relevant to your study. If you are not sure if a list item applies to your research, read the appropriate section before selecting a response.

Materials & experimental systems

n/a	Involved in the study
☒	☐ Antibodies
☒	☐ Eukaryotic cell lines
☒	☐ Palaeontology and archaeology
☒	☐ Animals and other organisms
☐	☒ Clinical data
☒	☐ Dual use research of concern
☒	☐ Plants

Methods

n/a	Involved in the study
☒	☐ ChIP-seq
☒	☐ Flow cytometry
☐	☒ MRI-based neuroimaging

Clinical data

Policy information about clinical studies

All manuscripts should comply with the ICMJEguidelines for publication of clinical research and a completedCONSORT checklist must be included with all submissions.

Clinical trial registration

The Adolescent Brain Cognitive Development (ABCD) study is not a clinical trial in the traditional sense. Instead, it is a longitudinal research study focused on understanding brain development and cognitive functioning in children and adolescents. While it shares some similarities with clinical trials, such as the collection of extensive data from participants, the primary goal of the ABCD study is not to test the efficacy of a specific treatment or intervention.

Study protocol

https://abcdstudy.org/

Data collection

The Adolescent Brain Cognitive Development (ABCD) study is a collaborative effort involving multiple data collection sites across the United States. These data collection sites play a crucial role in gathering comprehensive data from participants and contribute to the study's goals of understanding brain development in adolescents. Here is an overview of ABCD data collection sites: The ABCD study is conducted at numerous research institutions and universities across the United States. This multi-site approach enhances the study's ability to recruit a diverse and representative sample of participants. The data collection sites are strategically located in various regions, encompassing both urban and rural areas, to ensure that participants from different geographic backgrounds are included in the study. Each data collection site is equipped with state-of-the-art facilities for conducting assessments, interviews, and neuroimaging scans. These centers are designed to provide a comfortable and conducive environment for participants. Each site has a team of researchers, clinicians, and technicians trained to administer cognitive assessments, conduct interviews, and perform neuroimaging scans. These professionals follow standardized protocols to ensure consistency across sites. The data collection sites are responsible for coordinating and conducting follow-up visits with participants as they age. Each data collection site adheres to strict ethical guidelines and protocols to ensure the privacy, safety, and well-being of participants. Informed consent is obtained from both participants and their parents or guardians. The data collected at these sites are shared and analyzed collaboratively to facilitate a comprehensive understanding of adolescent brain development. Researchers from different sites work together to achieve the study's objectives.

Outcomes

The primary objective of the study was to investigate the effects of handedness on the asymmetry of brain connectivity and the laterality of motor connectivity pathways in different regions of the brain as a function of handedness in children aged 9 to 11 years. The secondary objective was to investigate association between handedness and the functional connectivity patterns of hand-motor areas while considering factors such as brain structure and connectivity. To assess brain functional connectivity, we tested for group differences in data-driven global functional connectivity density (gFCD) and hypothesis-driven seed-vertex correlation patterns using two, and one-sample t-test (2-sided). To assess structural connectivity, we used white matter diffusion metrics (fractional anisotropy, and mean, longitudinal, and transverse diffusivity). The studies on brain morphometry and myelination were based on cortical thickness, curvature, and sulcal depth metrics obtained from Tl weighted MRI scans and FreeSurfer segmentation. To assess brain asymmetry, we contrasted values in the right and left cortical hemispheres, independently for each metric. In summary, the study aims to provide insights into how handedness may influence the functional organization of the brain during childhood.

Plants

Seed stocks	n.a.
Novel plant genotypes	n.a.
Authentication	n.a.

Magnetic resonance imaging

Experimental design

Design type	resting-state
Design specifications	20 min
Behavioral performance measures	n.a.

Acquisition

Imaging type(s)	functional, structural, and diffusion.
Field strength	3T
Sequence & imaging parameters	3D Tlw inversion prepared RF-spoiled gradient echo and T2w variable flip angle fast spin echo pulse sequences with 1mm isotropic resolution were used for structural MRI. Multiband echo-planar imaging (EPI) with slice acceleration factor = 3 and 1.7mm isotropic resolution was used for diffusion MRI acquisition. T2*-weighted multiband EPI (TE/TR=30/800 ms, 2.4 mm isotropic resolution, 60 slices covering the entire brain, slice acceleration=6, and flip angle=52 degree) was used to acquire functional MRI (fMRI) data with blood-oxygen-level-dependent contrast.
Area of acquisition	Whole brain acquisition was used.

Diffusion MRI ☒ Used ☐ Not used

Parameters: b-values = 0, 500, 1000, 2000, and 3000 s/mm2, 96 diffusion directions; multi shell.

Preprocessing

Preprocessing software	Freesurfer (v5.3.0), FSL(v5.0.6), ABCD-BIDS pipeline.
Normalization	Volume-based spatial normalization to the MNI standard space was carried out through nonlinear registration using antsRegistration, employing brain-extracted versions of the Tlw reference and the ICBM 152 Nonlinear Asymmetrical template. A deformation field was used to correct the fMRI time series for susceptibility distortions.
Normalization template	MNI152
Noise and artifact removal	The fMRI pre-processing steps in the ABCD-BIDS pipeline perform standard denoising by regressing out time-varying head motion, white matter, and CSF signals, and the global signals that may impact group comparisons and separates fictitious motion induced by breathing-related magnetic field changes from true head motion.
Volume censoring	Image time points with framewise displacement>0.Smm were excluded from analysis.

Statistical modeling & inference

Model type and settings	Univariate analysis. Functional connectivity density mapping was based on a Pearson correlation threshold R>0.6 (first level) and random-effects (second-level) .
Effect(s) tested	Handedness

Specify type of analysis: ☐ Whole brain ☐ ROI-based ☒ Both

Anatomical location(s): The HCP2016 (379 cortical and subcortical gray matter partitions) and AtlasTrack (42 white matter partitions) probabilistic atlases were used.

Statistic type for inference<br>(See Eklund et al. 2016)	vertex-wise
Correction	FDR or Bonferroni corrections

Models & analysis

n/a	Involved in the study
[ ]	[x] Functional and/or effective connectivity
[x]	[ ] Graph analysis
[x]	[ ] Multivariate modeling or predictive analysis

Functional and/or effective connectivity	Pearson correlation.
